# Supplementary figures and images for: Sperm Toolbox—A selection of small molecules to study human spermatozoa
Source: PLoS One. 2024 Feb 20;19(2):e0297666. doi: 10.1371/journal.pone.0297666 (PMC10878532; doi:10.1371/journal.pone.0297666)

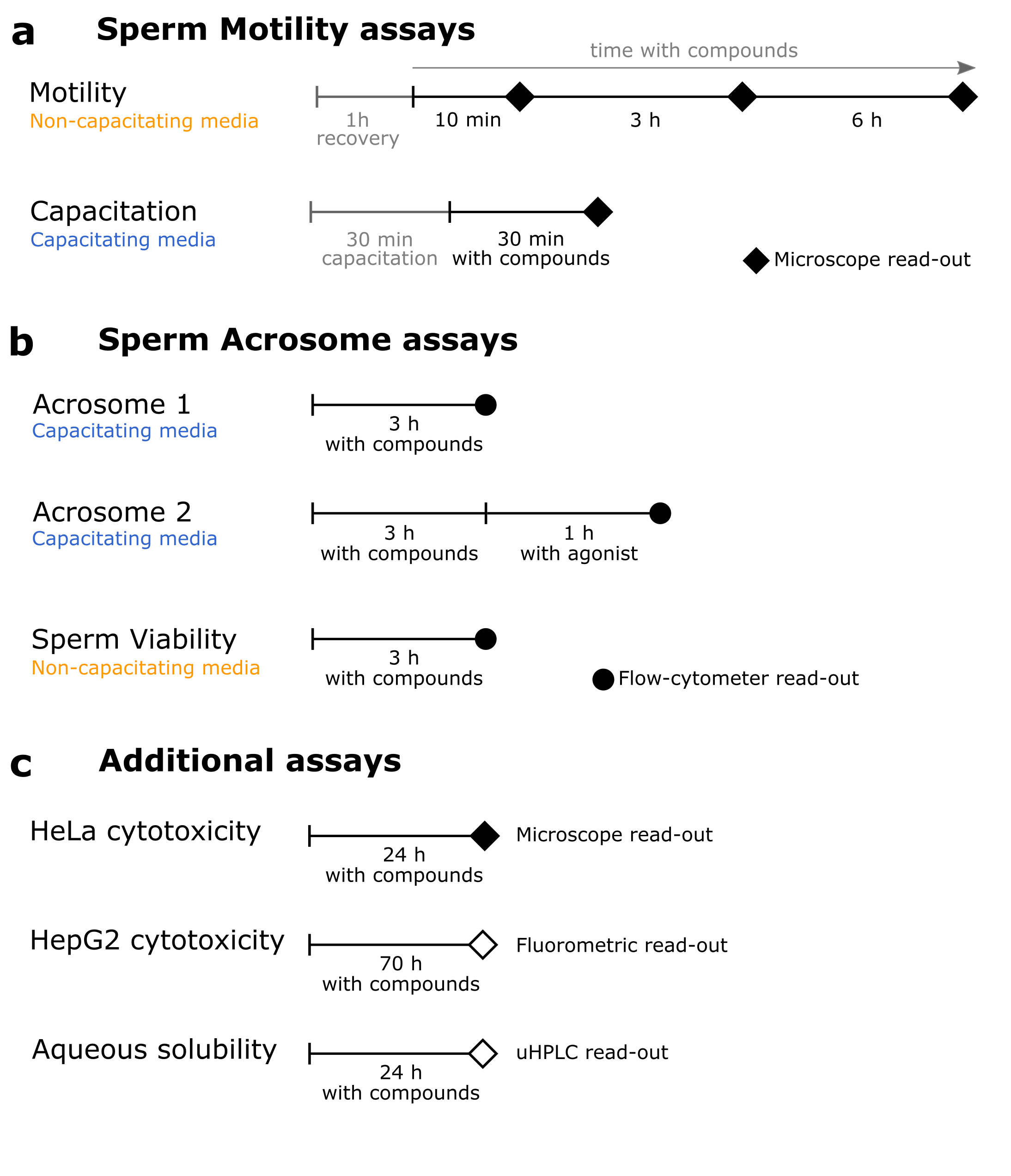

Supplement: S1 Fig — (a) Diagram showing sperm motility assays run on high-content microscope with compound incubation time/read-out time. Motility assays were run under non-capacitating conditions, with a 1 hour recovery phase after preparation of spermatozoa. Capacitation assays were run under capacitating conditions. Spermatozoa are capacitated for 30 min prior to compound incubation. (b) Diagram showing assays run on high-throughput flow cytometer to measure acrosome status and sperm viability. Incubation time with compound, agonist and read-out times are indicated. Acrosome assays run under capacitating conditions. Sperm viability assay run under non-capacitating condition. (c) Additional assays performed on sperm toolbox compounds. (TIF) [file pone.0297666.s001.tif]

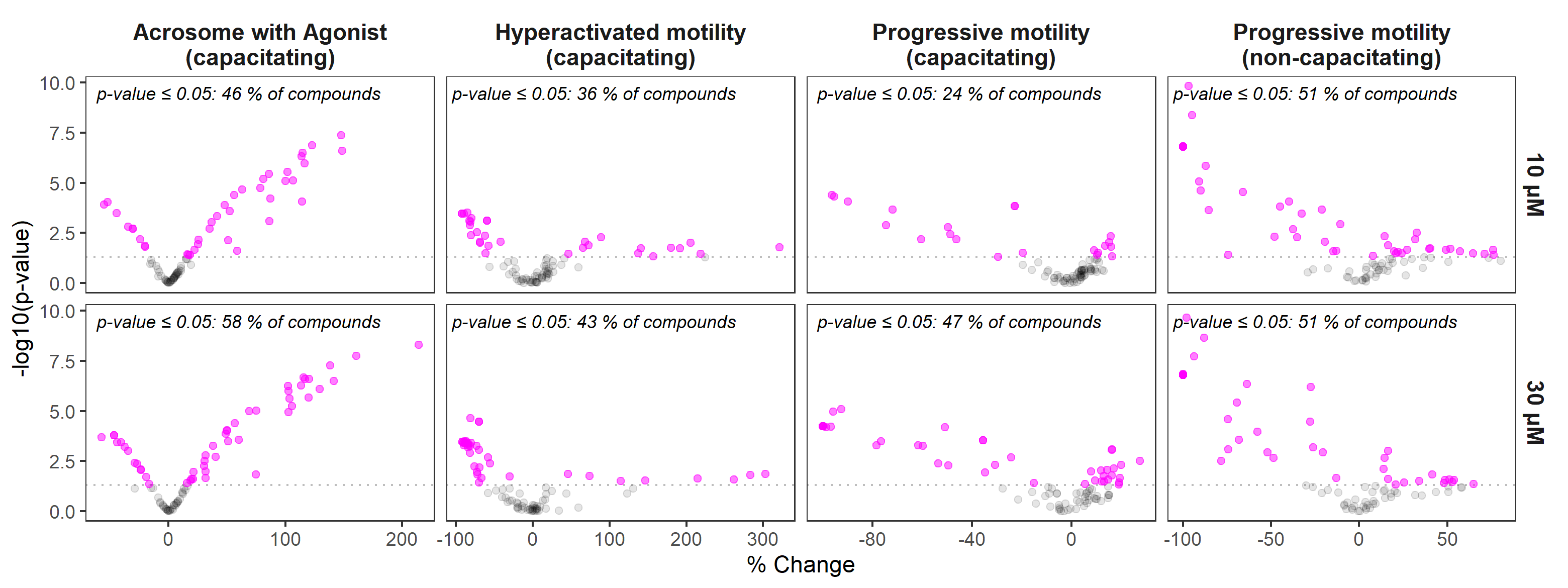

Supplement: S2 Fig — Upper panel indicates 10 μM, lower plot panels indicate 30 μM. Incubation times for assays are 3h with compound for Acrosome 2 assay and Motility (non-capacitating) (PM = progressive motility), and 30 min for the Capacitation assay (HA = hyperactive motility, PM = progressive motility). Dotted line indicates significance levels of 0.05. Any point above dotted line is indicated in magenta. Buffer conditions are indicated below Plot title (capacitating vs. non-capacitating). (TIF) [file pone.0297666.s002.tif]
