# Supplementary material for: Sperm Toolbox—A selection of small molecules to study human spermatozoa
Source: PLoS One. 2024 Feb 20;19(2):e0297666. doi: 10.1371/journal.pone.0297666 (PMC10878532; doi:10.1371/journal.pone.0297666)
Supplement: Attachment — Submitted filename: Response to Reviewers.docx [file pone.0297666.s004.docx]

Dear Dr Eldehna

Thank you very much for the reviews our paper describing the Sperm Toolbox as a resource for the research and drug discovery community. We appreciate the comments and insights from the reviewer.

We have separated each of the comments from the reviewers and provided responses in the following tables. We hope the comments and the accompanying ms edits satisfy the requests from the reviewers.

We look forward to your comments and responses.

For the authors,

Jason Swedlow

Comments from Editor

| Manuscript organization and presentation | The ms doc has been reformatted as directed in the formatting guidelines. |
| --- | --- |
| Funding Statement | We have removed funding info from the Acknowledgments. The Funding Statement should read: “The authors acknowledge support by the Bill & Melinda Gates Foundation (INV-007117). The funders had no role in study design, data collection and analysis, decision to publish, or preparation of the manuscript.” |
| Data deposition | Data associated with the results represented are  in Supplemental Info and now also deposited at zenodo with DOI: [10.5281/zenodo.10156887](https://zenodo.org/doi/10.5281/zenodo.10156887) |
| Captions in Supplementary Information | Added. |

Reviewer 1

| I recommend incorporating all tested compounds (with numbering) into the manuscript for enhanced readability. | We have added Table 1 that lists all compounds included in the Sperm Toolbox. |
| --- | --- |
| It would be beneficial to consolidate all results into tables based on their shared impact on sperm | This full table is available in S1 Data. We believe this is the correct location for the detailed data. However, we agree that a clearer presentation of the rationale for compound inclusion is necessary (Table 1) and a clearer presentation of at least some of data we collected (new Fig 2) is required and have addressed these requests. |
| The absence of microscope images depicting actual sperm alterations is noteworthy; their inclusion would enhance the study. | We have added a new Figure 2 that shows imaging and FACS-based results from selected compounds (note that our old Fig 2 is now Fig 3). This new figure, along with new Table 1, should make the Sperm Toolbox (STB) and our data more accessible to the reader. |
| The conclusion requires improvement as it lacks a concise summary of the main ideas. | We have substantially updated the Introduction and Conclusion. |

Reviewer 2

| Please clarify why 84 compounds only and what were the criteria of selection | We have substantially updated the Introduction and added Table 1 to provide more detail on rationale for compound inclusion in the STB. T |
| --- | --- |
| The authors should consider performing enzyme assay for the identified network targets or at least some structure based insilico studies | We agree that these steps will be important, but believe this should be taken forward in a dedicated follow-on study |
| Introduction is too short | We have substantially updated the Introduction and Conclusion. |
| The authors should discuss better the obtained results especially the importance of their findings and how it would help others | We have substantially updated the Introduction and Conclusion. |

Reviewer 3

| i) It is not particularly clear what the precise or relative value of the selected compound set is. And I would like to read some reflection on that because in the end the set is the deliverable that deserves reporting | We have substantially updated the Introduction and Conclusion. |
| --- | --- |
| Ii) the text lacked some introduction to the methods which made it harder to follow the reasoning and distinguishing observation from interpretation. | We have substantially updated the Introduction and Conclusion. |
| Various selection criteria were used to obtain the final set of screening compounds. To evaluate the relative power/value of this specific set it is important to evaluate the contribution of the individual compounds (i.e. could the set have been made smaller without losing much resolution) and at the same time the potential contribution of compounds that were left out (i.e. the resolution that still could have been gained). | Our criteria and the information we reviewed to establish the STB are now in Table 1 and examples of the data we collected are in our new Fig 2. |
| In that light, I have a conceptual problem with l 123 - 126 because the paper states that some included compounds hardly showed effects. Yet they were not removed because they were reported earlier in literature. But why then test this list of compounds at all, they were all already described in literature, as that was a selection criterion? And, at the other end, are all active compounds already known? Why have other compounds not been tested? Phrased in other words: Can the authors indicate the relative value of the panel at all, when following their line of reasoning? | This is now addressed in the text. We have elected to retain compounds in STB that were reported to have effects on sperm but that did not show strong activity in our assays precisely because 1) we believe our results will help others in the field decide whether to use these compounds in their own work and 2) we do not believe that our assays cover every aspect of sperm biology. We and others may develop additional assays that can be added to the STB assay library in the future. We will present these new data in future versions of the STB. |
| l 80 and following. It would help if the various tests were shortly introduced, maybe shortly describe the platform. Because such a short introduction to the available tests (within the existing platform, I assume) lacks, it is not really clear what the paragraphs are precisely about. e.g. l 95 starts with: "the acrosome assays" Given the text uptill then, the reader is not informed what the assay is about and why it is used. | We have substantially updated the Introduction, providing more detail on the assays used. |
| l 73 are -> were  l 74 indicate -> indicated; are -> were  l 77 consists -> consisted  l 83 consists -> consisted  l 85 and normalized -> What is meant here? Was the outcome compared to a control (substraction) or were they really normalized (division/multiplication). | All addressed. |
| in l 108 it is not clear whether it states an observation (then it should have been in past tense) or a conclusion (but then I would like to read some arguments). | We appreciate the point. The text has been substantially updated to clarify. |
